# Identifying MRI Markers Associated with Early Response following Laser Ablation for Neurological Disorders: Preliminary Findings

**DOI:** 10.1371/journal.pone.0114293

**Published:** 2014-12-11

**Authors:** Pallavi Tiwari, Shabbar Danish, Anant Madabhushi

**Affiliations:** 1 Department of Biomedical Engineering, Case Western Reserve University, Cleveland, Ohio, United States of America; 2 Division of Neurosurgery, Rutgers-RWJ Medical School, New Brunswick, New Jersey, United States of America; University of California, San Francisco, United States of America

## Abstract

There is a renewed interest in MR-guided laser interstitial thermal therapy (LITT) as a minimally invasive alternative to craniotomy for local treatment of various brain tumors and epilepsy. LITT allows for focused delivery of laser energy monitored in real time by MRI, for precise ablation of the lesion. Although highly promising, the long-term effects of laser ablation as a viable treatment option for neurological disorders have yet to be rigorously studied and quantified. In this work, we present a quantitative framework for monitoring per-voxel thermal-induced changes post-LITT over time on multi parametric MRI. We demonstrate that voxel-by-voxel quantification of MRI markers over time can enable a careful and accurate (a) characterization of early LITT-related changes (if and when they are exaggerated and when they subside), and (b) identification and monitoring of MRI markers that potentially allow for better quantification of response to LITT therapy. The framework was evaluated on two distinct cohorts of patients (GBM, epilepsy), who were monitored post-LITT at regular time-intervals via multi-parametric MRI. On a cohort of six GBM studies we found that (a) it may be important for the initial treatment-related changes to subside to more reliably capture MRI markers relating to tumor recurrence, and (b) T1w MRI and T2-GRE may better differentiate changes that may correspond to tumor recurrence from patients with no recurrence, as compared to T2w-MRI, and FLAIR. Similarly, our preliminary analysis of four epilepsy studies suggests that (a) early LITT changes (attributed to swelling, edema) appear to subside within 4-weeks post-LITT, and (b) ADC may be more reflective of early treatment changes (up to 1 month), while T1w may be more reflective of early delayed treatment changes (1 month, 3 months), while T2-w and T2-FLAIR appeared to be more sensitive to late treatment related changes (6-months post-LITT) compared to the other MRI protocols under evaluation.

## Introduction

There has been a recent interest in the potential utility of Magnetic Resonance Imaging (MRI)-guided Laser-induced interstitial thermal therapy (LITT) to treat brain tumors, such as glioblastoma multiforme (GBM) [1]–[3] and more recently, to treat epilepsy [4]. LITT potentially provides an advantage over other more invasive treatment options due to real-time image guidance, and the avoidance of larger incisions. Since LITT is based on thermal destruction of the target, it is not constrained by a maximum dose limit, and may be used opportunistically multiple times post- treatment if required [5]. Unlike other treatment options, the focused ablation via LITT may allow for a re-intervention (after the initial treatment) in patients who do not respond favorably to the treatment either due to negative treatment effects (such as disease recurrence) or incomplete treatment.

In the context of GBM, LITT is currently being explored for patients who, as determined by a multidisciplinary neuro-oncology board, are not candidates for standard-of-care open surgical debulking [1], [2]. Although promising, LITT is at a nascent stage as a viable treatment modality for GBM treatment. Only a few studies [6], [7] so far have attempted to characterize the underlying laser-tissue interactions due to LITT, as central coagulation necrosis and peripheral edema (early changes), and subsequent resorptive changes, the formation of a rim of granulation tissue as delayed-changes post-LITT [6]. However, to our knowledge currently there is relatively little information regarding the specific *in vivo* imaging characteristics of LITT-induced changes within and around the ablation zone for GBM patients.

**Figure 1:**
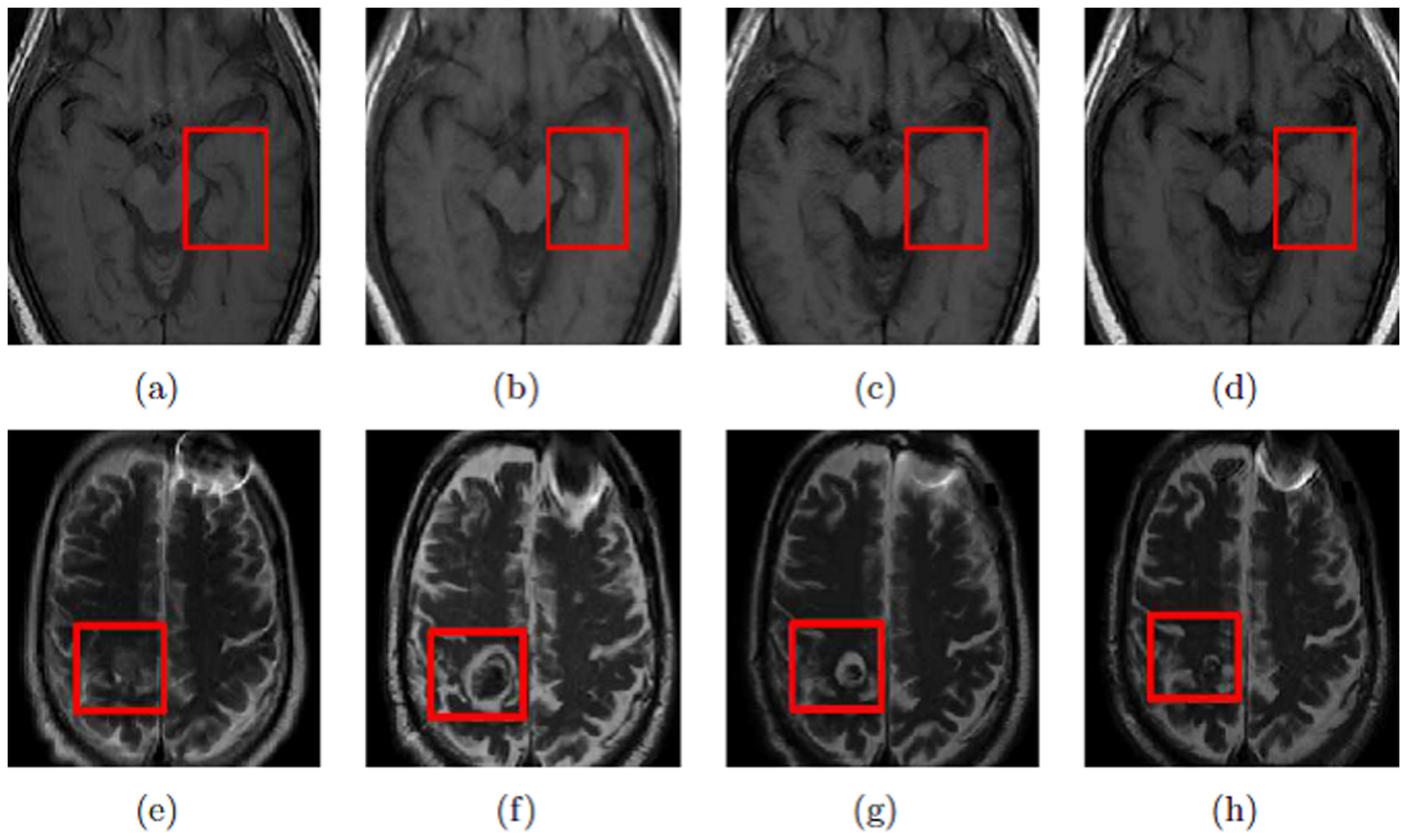
(a)–(d) show a 2D T1w MRI slice at different time points, pre-LITT (a), 24- hours post-LITT (b), 1-month post-LITT (c), and 3-month post-LITT (d) for a seizure-free epilepsy study, while 1(e)–(h) show a 2D T2w MRI slice at pre-LITT (e), 24-hours (f), 2-months (g), and 7 months (h) respectively for a successfully treated (no signs of recurrence at the time of evaluation) GBM study. Note how changes in imaging markers subside over time in the case of a LITT procedure with successful treatment.

More recently, LITT has also emerged as an alternative to traditional craniotomy for epilepsy, which attempts to ablate seizure focus with minimal damage to normal surrounding tissue [8]. In cases of lesional epilepsy, such as mesial temporal sclerosis (MTS), LITT can be used to ablate the focus under direct and real-time MRI monitoring [4]. Fig. 1a is an example of a sclerotic hippocampus that was targeted for ablation. The existence of imaging markers after ablation of epileptogenic foci can be explored in the context of treatment related changes (such as swelling, edema, seizure recurrence, and irreversible tissue damage) which present as early-, mid-, and delayed-effects [9], [10]. However, a quantitative study evaluating the changes in MRI imaging markers over time within the ablation zone to study LITT effects on epilepsy patients has not yet been performed.

Currently post-treatment changes for evaluating treatment response are monitored qualitatively via comparing volumetric changes of contrast enhancement on T1w MRI protocol acquired for follow-up (24-hours, 1-month, 3-months, 6-months post-treatment), with reference to pre-treatment T1w MRI (known as Recist criterion [11]). However, being a localized treatment modality, LITT is known to introduce local effects (such as swelling, edema, tissue necrosis) within the ablation zone. These early treatment related changes may be better reflected via a voxel-by-voxel analysis of changes in MRI markers monitored over time [12]–[14]. The regular monitoring of multi-parametric-MRI markers may provide us with insights on subtle changes in imaging markers that occur within the first few weeks due to the immediate effects of treatment, and potentially serve as surrogate markers for evaluating patient's response to treatment, potentially prior to changes in gadolinium enhancement, which is currently how local failure is evaluated. Additionally, per-voxel analysis of MRI markers [12]–[14] can be complemented with other standard tools, such as Recist criterion (volumetric changes) to better characterize response to LITT treatment using MRI.

The availability of multi-parametric-MRI protocols (structural, functional information available via T1w, T2w, GRE, FLAIR intensities, and apparent diffusion coefficient (ADC) values computed from diffusion weighted imaging (DWI)) acquired at multiple time points post-LITT (24-hours, 1-month, 3-months, 6-months, and so on) (Fig. 1) for follow-up, provides us with a unique opportunity to attempt to address the following types of questions in the context of evaluating post-LITT changes on imaging. For instance (1) can we monitor specific changes in MRI markers over time within the ablation zone for quantification of LITT changes? (2) can we identify which MRI marker is more sensitive to capturing treatment changes over time? (3) can we identify when confounding thermal effects within and around the ablation zone subside after LITT?

Researchers have previously investigated some of these questions in the context of evaluating response to treatment for various neurological disorders and cancer types by qualitatively following trends of changes in MR markers over time [3], [15]–[17]. However these investigations have used volumetric analysis along with image characteristics that are subjectively assessed and qualitatively defined and, therefore, potentially have inter-observer variability. Moreover, visual assessment of post-LITT MRI may not completely capture subtle localized changes within the ablation zone. Automated quantitative assessment may overcome inter-, and intra-observer variability introduced by visual inspection [18], [19] via the use of quantitative tools that can provide more reliable per-voxel quantitative assessment of changes in MR imaging markers post-LITT. However, one has to specifically account for the following challenges when developing a quantitative framework for evaluating LITT related changes,

- MR intensity non-standardness across pre- and post-LITT MRI: One of the major drawbacks of MRI is known to be the lack of a quantifiable (tissue specific) interpretation of image intensities [20]. MR images taken for the same patient on the same scanner at different times are known to appear different from each other due to a variety of scanner-dependent variations and, therefore, the absolute intensity values do not have a fixed meaning [21]. The intensities hence need to be aligned pre- and post-treatment for a per-voxel comparison so the MR images from different acquisitions have the same tissue-specific meaning.
- Accurate alignment of different MRI protocols and across pre-, and post-treatment at different time-points for computing voxel level absolute differences of the imaging markers (reflective of treatment changes): Accurate co-registration of MRI protocols (T1w, T2w, GRE, FLAIR, ADC) is essential to quantitatively compare changes in imaging markers on voxel-by-voxel basis, across different protocols, while accurate co-registration of MRI markers pre-, and at different time points post-LITT is required for careful monitor changes in MRI markers on a per-voxel basis over time, and
- A combination of MRI markers can capture treatment related changes over time with high sensitivity and specificity compared to a single MRI marker: A large volume of literature [22]–[28], including work in our group [23]–[26], has suggested that quantitative features fused across different MRI protocols provide more diagnostic information than what is available from a single protocol. There is hence a need for quantitative integration of different MRI markers that can capture complementary information across different MRI protocols and in turn potentially better reflect subtle treatment related changes on multi-parametric MRI.

**Figure 2:**
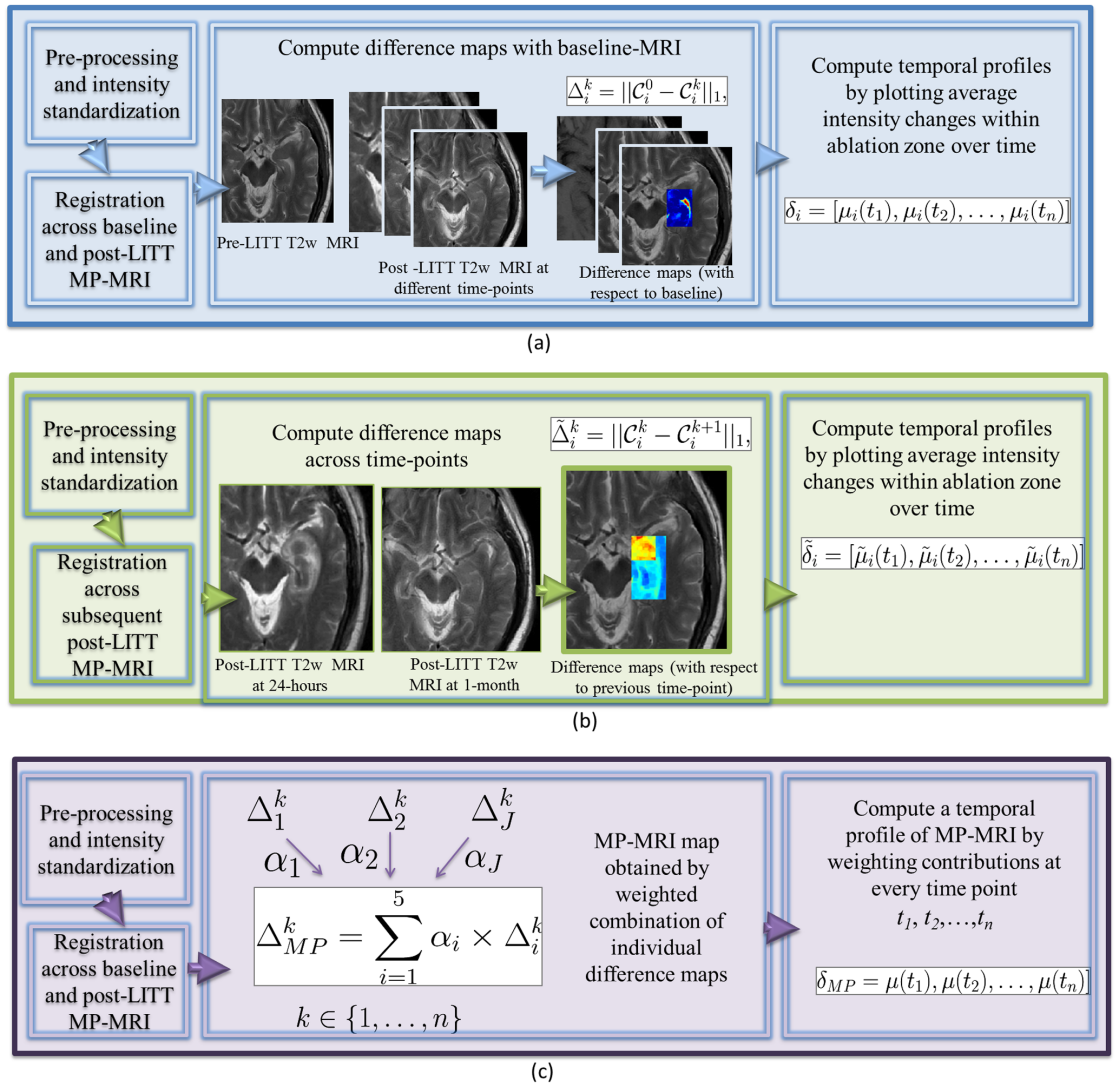
Flowchart showing different modules for each of the three objectives, where (a) illustrates the methods for objective 1 to obtain a temporal MRI profile of differences plotted with respect to baseline, while (b) illustrates the methods for objective 2 involving studying when early treatment changes subside by plotting differences in MRI markers at subsequent time-points. Fig. 2(c) illustrates the methods for objective 3 involving developing a fused multi parametric MRI signature by computing a weighted combination of MRI protocols at different time-points.

In this work, we address these challenges by developing a quantitative framework (Fig. 2) that encapsulates the appropriate intensity standardization, image co-registration, and quantification modules to accurately capture localized per-voxel treatment changes between pre-, and post-LITT, and weight them to create a multi-parametric MRI map that potentially can better capture treatment related changes than any of the individual MRI protocols. Below we summarize each of the different modules of the presented quantitative framework.

**Module 1. Correction of MRI marker drift**: Pre-and post-LITT MR markers (T1-w, T2-w, GRE, FLAIR intensities) will be quantitated by correcting for intensity drift between acquisitions using intensity standardization developed by Nyul and Udupa [20] to ensure intensities, when compared on a per-voxel basis, have the same tissue specific meaning.

**Module 2. Registration**: An affine co-registration scheme (linear transformation involving rotation, translation, scaling, and shear) [29] is employed for alignment of different MRI protocols, as well as for pre-, and post-LITT MRI at different time-points for evaluating LITT response. Since LITT is a focal treatment, changes due to the treatment will tend to be localized around the target of interest, and an affine co-registration scheme should be sufficient to align pre-, and post imaging at different time-points, instead of a sensitive non-rigid co-registration scheme (such as a basis-spline involving higher order non-linear transformations to register images) which may lead to spurious co-registration.

**Module 3. Quantification of differences in MR intensities between pre, and multiple post-LITT time points**: A difference map is computed between pre-LITT MRI markers (T2-weighted intensities, T1-weighted intensities, GRE intensities, FLAIR intensities and ADC values), and the corresponding post-LITT MRI marker at every time-point, to capture and monitor subtle changes in MRI markers on a per-voxel basis. The changes in MRI markers monitored over time are used to quantify the relative importance of individual MRI markers in capturing treatment related changes over time.

**Module 4. Creating a multi-parametric MRI map by fusing difference maps obtained for different MRI protocols**: A multi-parametric map is created by combining pre-, and post-MRI intensity difference maps. The combination consists of linear weighting of difference maps of MRI markers based on how responsive they are (computed in Module 3) in accurately capturing treatment related changes over time.

We evaluate our quantitative framework to understand and monitor treatment changes over time for two disease types, epilepsy and GBM. Although we realize that the MRI changes for epilepsy and GBM are inherently different as different tissues are targeted during treatment (tumor in the case of GBM, and hippocampus for epilepsy), the purpose of this study is to provide a quantitative framework for monitoring changes in MR markers over time that can be used to capture LITT related changes over time. It is our intent that the analytic platform will pave the way for building novel imaging-based predictors of LITT response in neuroimaging disorders (including GBM and epilepsy), and thus enable improved understanding of patient's response to LITT.

## Materials and Methods

### Pre-processing of pre-, and post-LITT multi-parametric MRI

**Figure 3:**
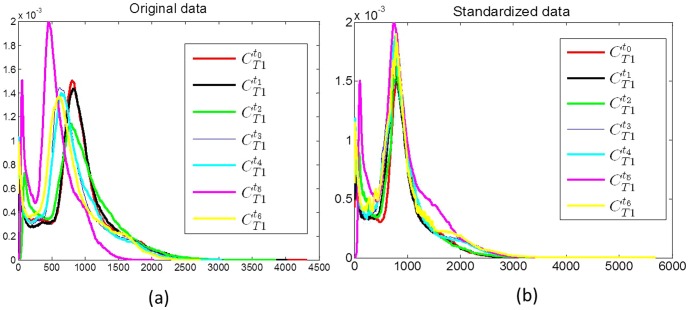
Illustration of intensity drift between pre- (red) and post-LITT at different time points (shown in blue, green, yellow, magenta, and cyan) for T1w MRI, by plotting the corresponding distributions along the same axis. Note that after intensity standardization, the distributions across different time-points are no longer misaligned, suggesting successful correction of the drift artifact.

When the histograms for T1w MRI at 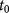 (red) and at different time-points 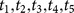 (blue, green, yellow, magenta, cyan respectively) are plotted together (Fig. 3a)), it is clear they have different intensity ranges and are not in alignment. In order to quantitatively compare the changes in MRI markers between pre- and post-LITT acquisitions, an intensity standardization scheme developed by Nyul and Udupa [20] was implemented in-house using a Matlab software package and was used to automatically identify corresponding landmarks on each of the histograms, and subsequently non-linearly map them to one other. As a result of intensity standardization, the histograms are aligned (Fig. 3(b)) and the MRI markers can be directly compared across different time-points. Intensity standardization was performed for corresponding pairs of MRI markers (T1w, T2w, GRE, FLAIR intensities) between pre- and post-LITT MRI acquisitions at different time-points. Since ADC is a standardized quantitative measure (obtained from DWI) across acquisitions, no intensity standardization was performed for ADC images.

### Co-registration of multi-parametric MRI between baseline-MRI and across different time-points

**Figure 4:**
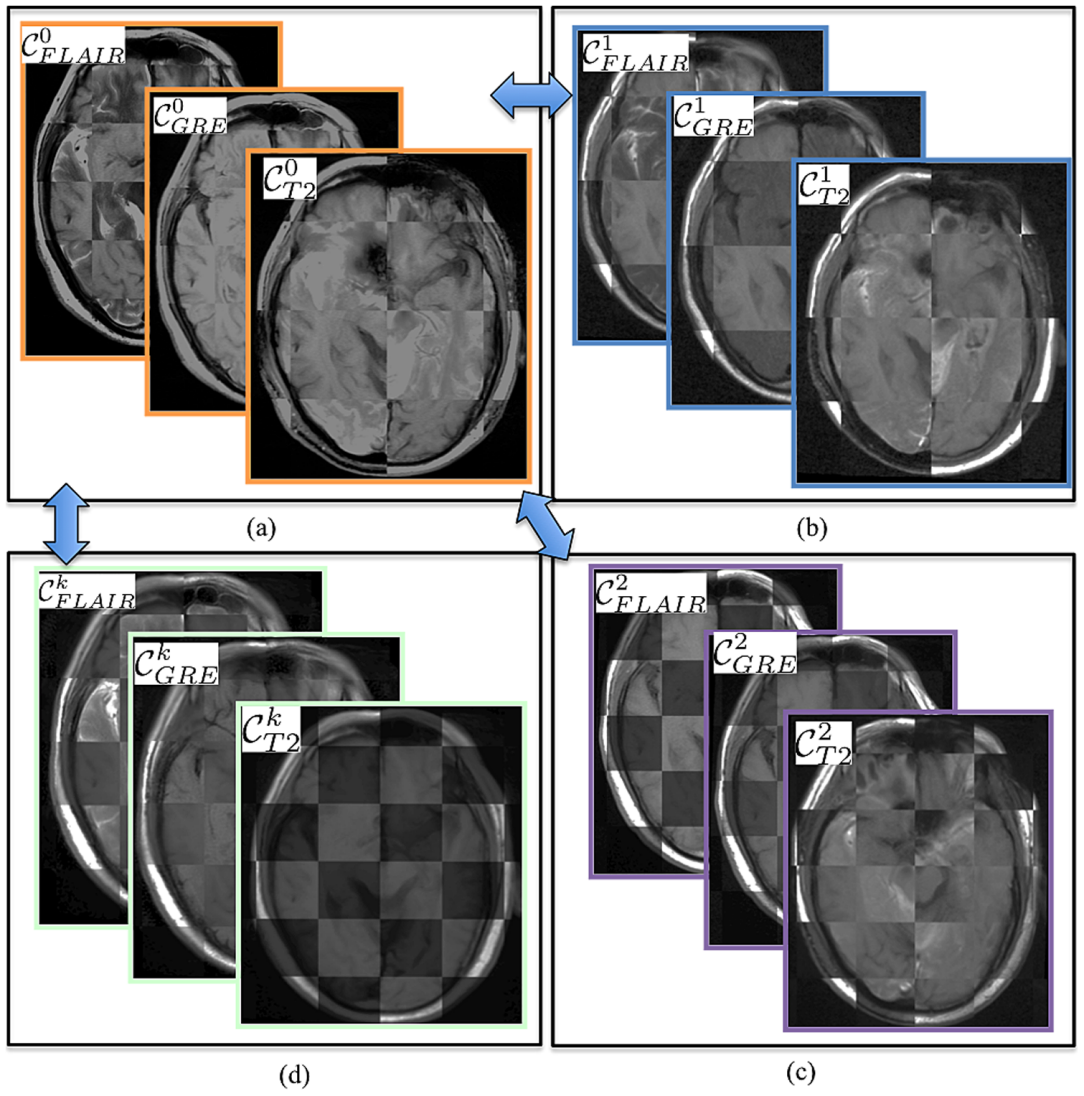
Checker-box representation of co-registration across different MR sequences at different time-points with respect to pre-LITT T1w MRI for (a) different pre-LITT MRI acquisitions, (b) 24-hour post-LITT MRI acquisitions, (c) 1-month post-LITT MRI acquisitions, and (d) 3-month post-LITT MRI acquisitions. Note the perfect alignment of the boundaries across different protocols and time-points. Pair-wise co-registration with respect to one reference MR sequence (T1w MRI) allows for quantitative comparison of different protocols at different time-points.

A 3D affine transformation [29] with 12 degrees of freedom, encoding rotation, translation, shear, and scale, was employed to accurately align post-LITT MRI with reference to pre-LITT (at time-point 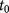) T1w MRI, 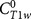, which yielded a registered 3D MRI volume, 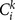 at every time-point 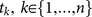, 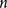 being the total number of time points evaluated post-LITT, for every MRI protocol, 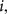 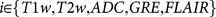. Since MRIs are acquired at different time-points, the number of slices for different MRI protocols and resolution across acquisition may be different across pre-, and post-LITT MRI protocols. Hence, during co-registration, the 3D volume is appropriately resampled and interpolated, to account for varying voxel sizes and resolutions between different time-points and protocols. Note that all the different MP-MRI acquisitions are aligned to the pre-treatment frame of reference pre-LITT T1-w MRI to enable per-voxel quantitative comparisons across different time-points and protocols post-LITT (Fig. 4).

### Objective 1: Evaluating temporal profiles of changes in MRI markers over-time

#### Step 1: Alignment of multi-parametric MRI between baseline-MRI and across different time-points

Post-LITT MRI at every time-point is aligned with reference to pre-LITT MRI, for every MRI protocol, T1w, T2w, GRE, FLAIR, ADC using an affine-based co-registration scheme (described above). The co-registration with reference to pre-LITT MRI allows for alignment of all protocols across different time-points in the same frame of reference for a voxel-by-voxel comparison.

#### Step 2: Generating difference maps for each time point with respect to baseline-MRI for individual protocols

The range of values for pre and post-LITT MRI was normalized to have a mean of 0 and a mean absolute deviation of 1. This ensured that the different MR parameter values were in a comparable range of values when quantifying differences across pre- and post-LITT MRI. An ablation zone, 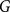 is then identified on pre-LITT T1w MRI, and is used to compute a differences map 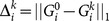 of absolute differences of MRI markers for every time-point 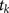 with reference to pre-LITT MRI on a per-pixel basis. Computing absolute differences allowed for quantification of absolute changes in imaging markers across different time-points, with respect to baseline (pre-LITT) MRI for every MRI protocol.

Each of the difference maps, 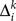, where 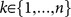 and 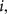 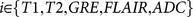 can be visualized by utilizing a color map, such that blue corresponds to small difference values and red corresponds to areas of high differences. Therefore, regions corresponding to large changes within the ablation zone should be highlighted by red on the difference map. Along similar lines, regions corresponding to little to no change in the ablation zone should be highlighted in blue on the difference map. Each difference map is implicitly normalized between 0 and 1, where 1 corresponds to a large difference and 0 corresponds to no difference (between the pre- and post-LITT MRI).

#### Step 3: Extracting a temporal profile for capturing MP-MRI differences across time-points with baseline-MRI

The median intensity is computed within the ablation zone of the difference maps for every MRI protocol. Median provides a measure of the most commonly occurring values within a range of values, and was used to quantify the most commonly occurring intensity changes in MRI markers within the ablation zone. A higher median value would hence reflect a high change in intensity values (between pre- and post-LITT MRI) while a low value would reflect a small change in intensity values within the ablation zone. We then extract a temporal MRI profile for every MRI protocol 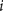 as 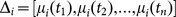, by plotting median intensity 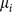 at every time-point 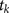 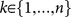. The temporal profile reflects the trend of changes in MR intensities over time, and allows for quantification of changes that occur post-LITT at different time-points.

### Objective 2: Identifying post-LITT MRI markers that are more sensitive to capturing treatment changes over time

#### Step 1: Alignment of MP-MRI protocols across subsequent time-points

Post-LITT MRI at every time-point is registered with reference to pre-LITT T1w MRI, for every MRI protocol using the methods illustrated in the co-registration Section. The co-registration with reference to pre-LITT T1w MRI allows for alignment of all protocols across different time-points in the same frame of reference for a voxel-by-voxel comparison.

#### Step 2: Generating difference maps for individual protocols

A differences map is computed within the ablation zone as an absolute difference of imaging markers as, 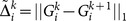, where 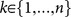, is the time point post-LITT, 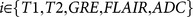 is the imaging protocol evaluated. Individual MRI parameter difference maps allow for quantification of changes in imaging markers across subsequent time-points, and can be visualized via a color map representation as detailed in Module 2 of Objective 1, such that blue corresponds to small difference values and red corresponds to areas of high difference. Each difference map, 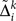, is implicitly normalized between 0 and 1, where 1 corresponds to a large difference and 0 corresponds to no difference (between subsequent post-LITT MRI).

#### Step 3: Extracting temporal profiles of MRI markers

The median intensity is computed within the ablation zone of the computed difference maps for every MRI protocol. We then extract a temporal MRI profile for every MRI protocol 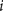 as 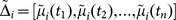, by plotting 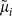 at every time-point, 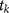, 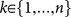.

### Objective 3: Identifying appropriate time-point when LITT induced changes dissipate for assessment of treatment efficacy

#### Step 1: Alignment of multi-parametric MRI across time points

For a specific time-point, each of post-LITT MRI protocol is individually aligned to pre-LITT T1w MRI via an affine co-registration scheme as described in the co-registration Section to align all MRI protocols across multiple time-points to a common frame of reference. The co-registration with reference to pre-LITT T1w MRI allows for alignment of all protocols across different time-points in the same frame of reference for a voxel-by-voxel comparison.

#### Step 2: Generating a multi-parametric weighted MRI map

The relative contribution of each protocol is obtained via a difference map by identifying MRI markers that change most dramatically post-LITT and hence may serve as candidate MR markers that better capture treatment related changes post-LITT. The individual contributions are obtained for each protocol depending on their ability in differentiating positive and negative treatment related changes, and are combined to develop a fused MP-MRI marker that simultaneously captures the discriminability across all individual MRI markers, and may potentially serve as a surrogate marker to distinguish successful treatment from tumor recurrence.

Once a difference map is computed within the ablation zone as an absolute difference of image intensity markers between pre- and post-LITT MRI, a fused MP-MRI marker map is obtained by leveraging difference maps for each of the five MRI markers (T1w, T2w, GRE, FLAIR intensities, and ADC values) as 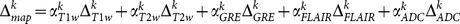, where 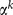 weights the relative contribution of each of the individual imaging markers at every time point, 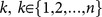.

#### Step 3: Extracting a fused MP-MRI temporal profile that captures combined differences of imaging markers with respect to baseline-MRI

For a specific protocol, 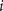, the median intensity value 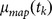 is computed within the ablation zone, 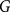. We then extract a fused temporal MRI profile as 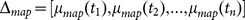, by plotting 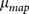 at every time-point 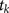, 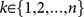. The fused temporal profile characterizes an optimized weighted sum of trends of changes across different MRI profiles that maximizes separation between successful treatment and tumor recurrence, and hence may serve as an improved predictor for patient's response to treatment.

### Implementation details

The co-registration across different MRI sequences was implemented via the 3D Slicer software 4.1. (http://www.slicer.org/). Intensity standardization as well as modules 2 and 3 for each of the three objectives involving computation of difference maps, development of temporal signatures, optimization and integration of weights across different MRI protocols was implemented via the Matlab software package.

The optimal weights for each of the individual MRI protocols were obtained via a grid search strategy [30]. The weights for each of the 5 protocols were varied between 0 and 1 in the increments of 0.2, such that 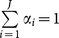, which resulted in a total of 125 weighted combinations of MP-MRI profiles. The top 20% of the 125 MP-MRI profiles that changed most drastically over time were selected (yielding 25 candidate profiles) such that changes in difference maps are optimized across time-points to a patient's response to treatment. A voting scheme was then implemented to determine the protocols that occurred most frequently across the 25 candidate profiles, and the weight for each protocol (normalized between 0 and 1) was assigned based on its frequency of occurrence across the 25 profiles, at each time-point.

### Dataset Description

An FDA-cleared surgical laser ablation system (Visualase Thermal Therapy System; Visualase, Inc., Houston, TX) was employed for all LITT procedures. Details on available protocols and time-points for the two cohorts of datasets, epilepsy, and GBM are provided in Table 1.

**Table 1:** Table demonstrating the two datasets and available protocols for follow-up MRIs for the four epilepsy studies and six GBM studies under evaluation.

Dataset	MRI Protocols	MRI follow-ups	Status
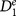	T1w, T2w, ADC, GRE, FLAIR	24-hrs, 1, 3, 6-months	*S*
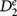	T1w, T2w, ADC, GRE, FLAIR	24-hrs, 2, 6-months	*S*
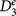	T1w, T2w, ADC, GRE, FLAIR	24-hrs, 1-month	*S*
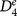	T1w, T2w, ADC, GRE, FLAIR	24-hrs, 1-month	*S*
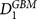	T1w, FLAIR	24-hrs, 3 months	*S*
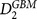	T1w, FLAIR	48-hrs, 1-month	*S*
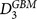	T1w, T2w, ADC, GRE, FLAIR	24-hrs, 2, 4, 7, 9, 11-months	*S*
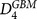	T1w, T2w, ADC, GRE, FLAIR	24-hrs, 3, 4, 7, 8-months	*S*
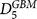	T1w, T2w, ADC, GRE, FLAIR	24-hrs, 2, 4, 6, 8, 11-months	*F*
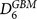	T2w, GRE, FLAIR	1, 5, 7-months	*F*

The two cohorts were collected as a part of an ongoing IRB approved study "Prospective and Retrospective Database Review of Magnetic Resonance Thermometry Guided Laser Induced Thermal Therapy (LITT)" (under IRB protocol #0220110296), at Robert Wood Johnson Hospital, New Jersey. Written consent was obtained from all patients for long-term follow up. Secondary analysis was performed on the anonymized data derived from data collected under informed consent. Since de-identified data was used for analysis, IRB consent was not required.

#### Dataset 1: Epilepsy studies

Four epilepsy patients were monitored post-LITT via MP-MRI (T1w, T2w, GRE, FLAIR, and ADC) as a part of an ongoing study at Rutgers-RWJ Medical School between 2011-2013, after initial 1.5-Tesla multi-parametric MRI. Post-LITT, patient 1 was reimaged after 24-hours, 1-month, 3-months, and 6-months while patient 2 was reimaged after 24-hours, 2-months, and 6-months. Patients 3 and 4 only had short-term follow up available and were reimaged at 24-hours and 1-month.

A scheme proposed by Engel [31] (de facto standard when evaluating postoperative outcomes for epilepsy surgery) that categorized epilepsy outcomes as, free of disabling seizures (class I), rare disabling seizures (“almost seizure-free”) (class II), worthwhile improvement (class III), and no worthwhile improvement (class IV), was used to obtain patient outcomes. All four patients were identified as Class I Engel outcome (seizure freedom (***S***)).

#### Dataset 2: GBM studies

Six GBM patients were monitored post-LITT via MP-MRI (T1w, T2w, GRE, FLAIR, and ADC) as a part of an ongoing study at Rutgers-RWJ Medical School between 2009-2013, after initial 1.5-Tesla MP-MRI. Post-LITT, patients were imaged at different time-points depending on their post-operative condition and availability post-LITT. Two of the six patients had tumor recurrence within one-year of LITT treatment, and hence identified as having tumor recurrence (***F***). The remaining four were identified as successful treatment (***S***).

### Surgical Procedure

The details of the surgical procedure have been previously described [16]. Briefly, the procedure is performed in the following manner. The appropriate entry point and trajectory angle are identified using the Medtronics Stealth S7 (Medtronics, Inc., Minneapolis, MN) using merged MRI and CT images. A stab incision is made at the entry site, followed by the creation of a burr hole using a handheld 3.2 mm twist drill. The Visualase Thermal Therapy System (Visualase, Inc., Houston, TX) bone anchor is placed using the alignment rod and precision aiming device, and then secured to the calvarium. The laser catheter is introduced through the fixed bone anchor. The laser used in the Visualase system is a 15-W, 980-nm diode laser, flexible diffusing tipped fiber optic, and 17-gauge internally cooled catheter. The patient is transferred to the MRI suite for the remainder of the procedure. In the MRI suite, the Visualase hardware system is connected to the MRI scanner, which allows for real-time thermal monitoring. The delivery of a test dose (typically, 3 W, 10–20 seconds), gives confirmation of laser placement. Safety margins are planned using the Visualase software such that the surrounding tissue's temperatures do not surpass the predetermined limit (50°C) and the center of the lesion does not exceed 90°C (to avoid steam vaporization). In addition, the software allows for target temperature placement in multiple orthogonal planes if necessary based on the lesion location. Laser treatment is then started at the appropriate dose and duration to achieve maximal lesion destruction. During the ablation procedure, the software imports thermal imaging information every 5 seconds (for single plane thermal imaging), allowing real-time analysis of the ablation process. There were no partial treatments in this series. Most patients were discharged within 24–36 hours in the absence of complications or other general medical conditions.

## Results

In this section, we detail our findings while evaluating our framework in the context of these objectives: (1) evaluating temporal profiles of changes in MRI markers to quantify changes post-LITT over-time, (2) identifying post-LITT MRI markers that are more sensitive to capturing treatment changes over time, and (3) evaluate when early LITT induced changes dissipate over time. Each of the experiments below corresponds to each of these three objectives and how they relate to evaluating response to LITT treatment in epilepsy and GBM patient studies.

### Experiment 1: Evaluating the framework to assess temporal profiles of changes in MRI markers over-time

**Figure 5:**
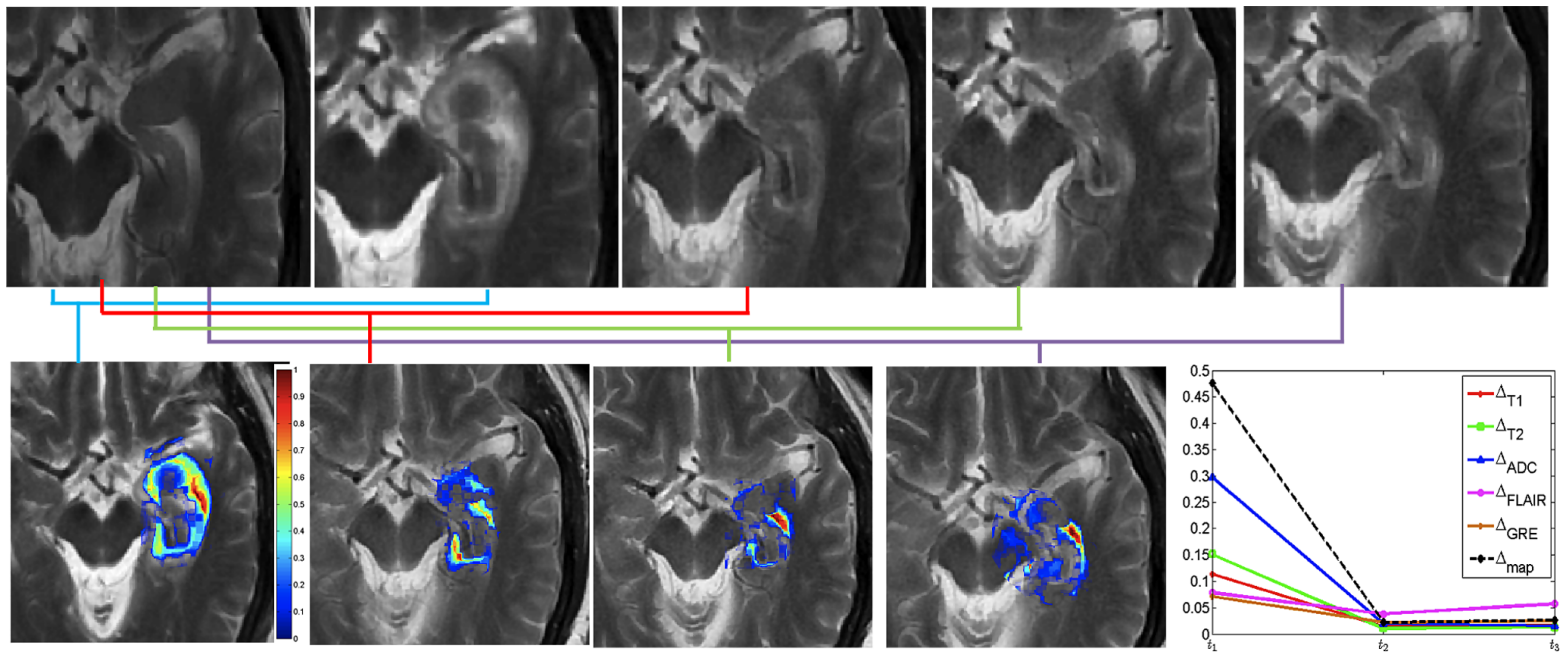
Original T2-w MRI images for (a) baseline (pre-LITT), (b) 24-hour, (c) 1-month, (d) 3-month, (e) 6 month post-LITT. Figs. 5(f) 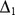, (g) 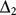, (h) 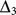, and (i) 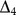 correspond to difference maps for T2-w MRI acquired at each of 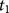, 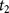, 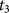, and 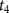 with respect to 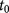. Fig. 5(j) shows temporal profiles of every MR protocol 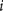, 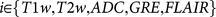 reflecting the changes in imaging markers at different time-points with respect to baseline scan.

Fig. 5 shows changes across T2-w MRI intensities at different time-points with respect to baseline-MRI for an epilepsy study. The original T2-w MRI images for baseline (pre-LITT), 24-hour, 1-month, 3-months, and 6 months are shown in Figs. 5 (a), (b), (c), (d), and (e) respectively, while the difference maps with respect to baseline for each of 24-hour, 1-month, 3-months, and 6-months are shown in Figs. 5 (f), (g), and (h) respectively. Mean intensity for every difference map is obtained within the epileptogenic focus for every protocol, T1w, T2w, GRE, FLAIR, and ADC and recorded over different time-points. Fig. 5(j) shows different temporal profiles (normalized between 0 and 1) created for each of the protocols for one patient study obtained by plotting the mean intensity value within the difference map at every time-point. Fig. 5 further suggests that, (a) intensity differences consistently decrease over all protocols for patients with successful treatment, and (b) the intensity differences after 1-month are considerably reduced as compared to that within the first 1-month which suggests that thermally induced changes to benign regions due to LITT occur within the first-month post-LITT.

**Figure 6:**
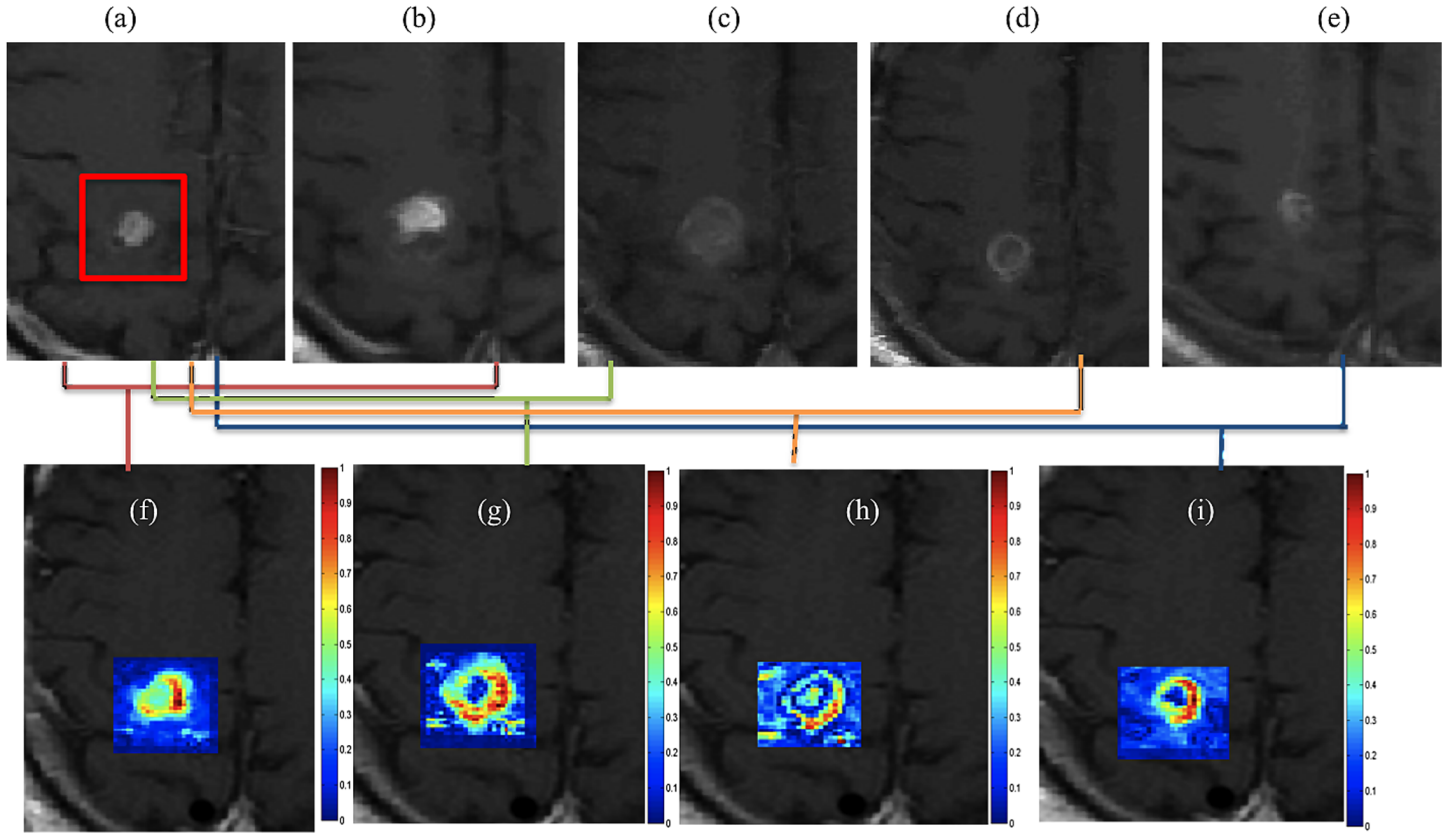
Original T2-w MRI images for (a) baseline (pre-LITT), (b) 24-hour, (c) 1-month, (d) 3-month, (e) 6 month post-LITT. Figs. 6(f) 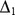, (g) 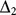, (h) 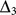, and (i) 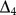 correspond to difference maps for T2-w MRI acquired at each of 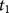, 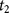, 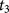, and 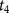 with respect to 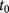. Fig. 6(j) shows temporal profiles of every MR protocol 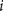, 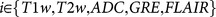, reflecting the changes in imaging markers at different time-points with respect to baseline scan.

Fig. 6(a) shows a pre-LITT T1w MRI image, while Figs. 6(b)–(e) show multiple time-points post-LITT for time-points at 24-hours, 2-months, 4-months, 7-months, and 11-months respectively for a GBM study identified as successful treatment. The corresponding color maps for time-point 1 to time-point 4 with respect to pre-LITT T1w MRI are shown in Figs. 6(f), (g), (h), and (g) respectively. Fig. 6 seems to suggest that (a) changes in MR parameters are enhanced at 24-hours post-LITT, and (b) intensity values decrease across time-points post-LITT after initial enhancement (at 24-hours) for successful treatment.

### Experiment 2: Evaluating the framework to study when early LITT induced changes have subsided allowing for assessment of treatment efficacy

**Figure 7:**
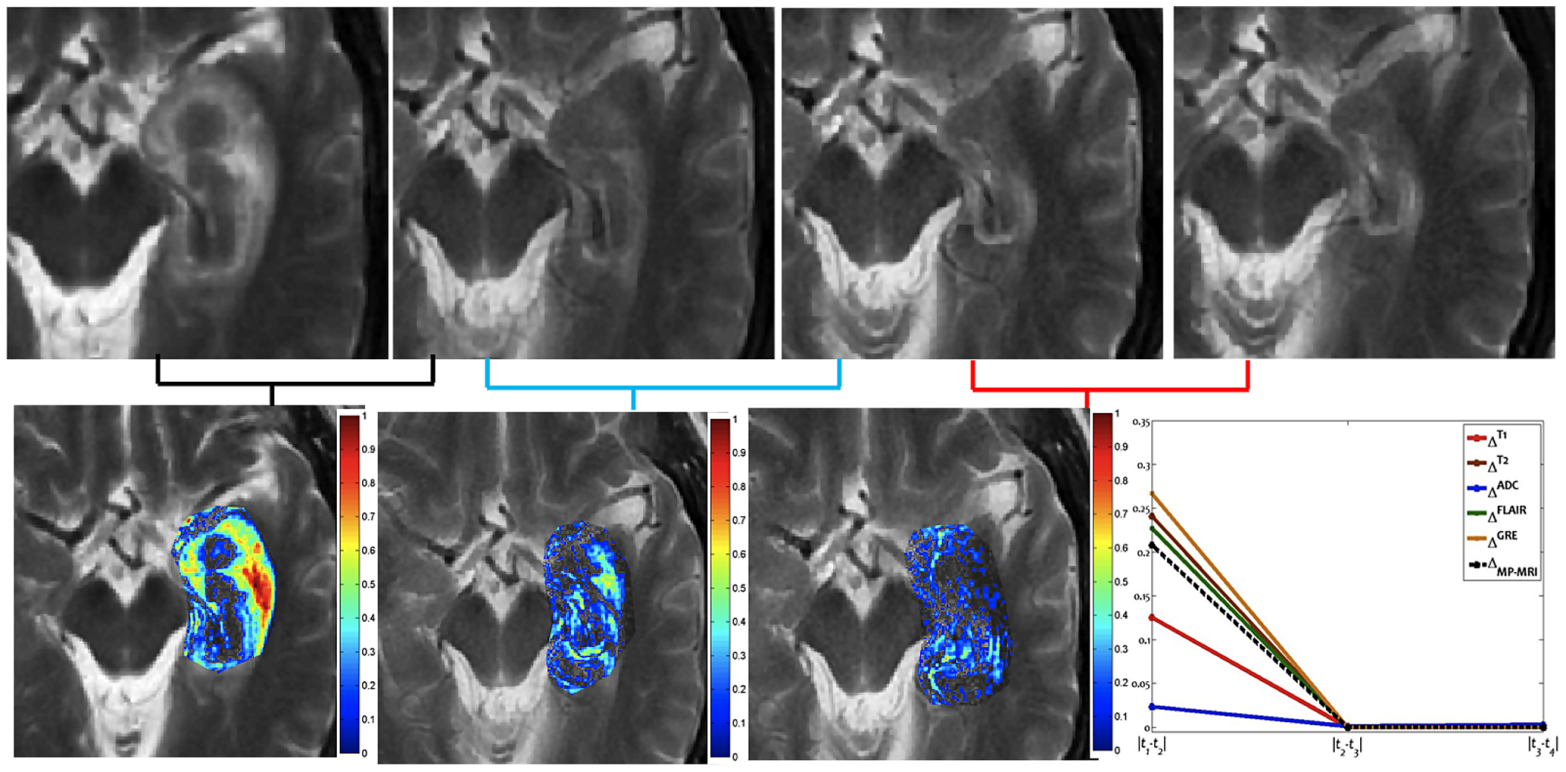
Original T2-w MRI images for (a) 24-hour, (b) 1-month, (c) 3-month, and (d) 6 month post-LITT. (e) 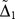, (f) 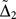, and (g) 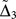, corresponding to difference maps for T2-w MRI computed across subsequent time-points 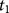, 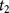, 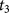, and 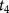. Fig. 7(h) shows temporal profiles of every MR protocol 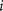, 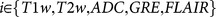 reflecting the changes in MR markers at subsequent time-points.

Fig. 7 shows difference maps obtained across multiple time-points for the T2-w MRI protocol for an epilepsy study. Figs. 7 (a)–(d) show the original T2-w MRI slices containing the ablation zone for 24-hours, 1-month, 3-months, and 6-months respectively, while the corresponding difference maps, 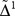, 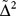, and 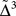 are shown in Figs. 7 (e), (f), and (g) respectively. Temporal profiles for every protocol, T1w, T2w, GRE, FLAIR, and ADC are shown in different colors in Fig. 7(h), obtained by plotting the mean intensity value at every time-point. Note how the changes are more prominent between 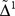 as compared to 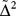, and 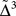. This appears to suggest that most of the early LITT effects such as edema, and swelling for epilepsy treatment occur and subside within the first one month.

**Figure 8:**
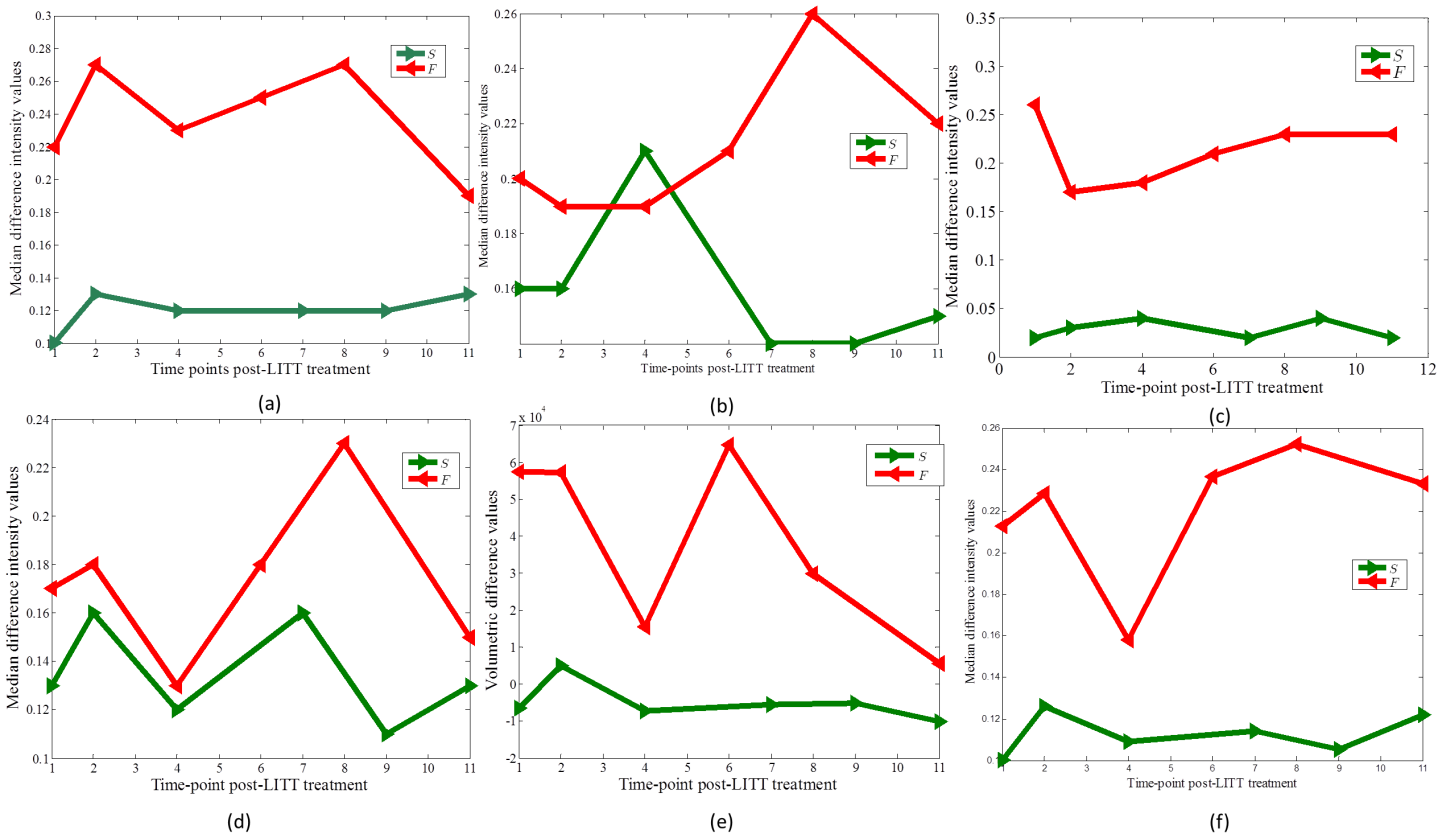
Temporal profiles showing changes in MRI markers over time for (a) T1w MRI, (b) T2w MRI, (c) T2-GRE, (d) FLAIR, (e) volumetric changes on T1w MRI, and (f) fused multi-parametric MRI for a GBM patient identified as responder (shown in green), against a GBM patient identified as a non-responder (shown in red) to the LITT treatment. Note that multi-parametric MRI profile provide a better distinction across the two classes as compared to individual T1w, T2w, and T2-GRE temporal profiles, and shows similar trends as obtained via volumetric analysis post-LITT.

Fig. 8 shows temporal profiles computed for T1w MRI (a), T2w MRI (b), T2-GRE (c), and T2-FLAIR (d) respectively, for a GBM patient identified to have successful treatment, no recurrence at the time of evaluation, (shown in green), against a GBM patient identified to have tumor recurrence (shown in red) to the LITT treatment. The trends of changes across the two classes suggest that, (a) MR changes for successful treatment become steady after a brief spike (due to edema and swelling) as against the patients with tumor recurrence where the trends are not consistent, and (b) 16-week (4-month post-LITT) time-point for T1w, T2w, and T2-GRE MRI, appears to show a better distinction between successful treatment (no signs of recurrence) and tumor recurrence than T2-FLAIR. For each of these protocols (T1w, T2w and GRE), changes in imaging markers for successful treatment become steady after 4-months, expect for tumor recurrence, where the difference intensities start to spike.

### Experiment 3: Evaluating the framework to identify post-LITT MRI markers that are more sensitive to capturing treatment changes over time

**Figure 9:**
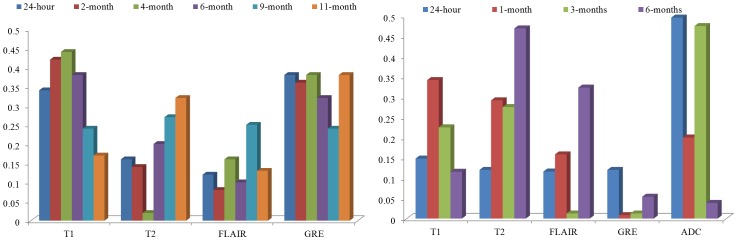
Contributions of each of the MR protocols (normalized between 0 and 1) in (a) capturing treatment changes post-LITT at 24-hour, 1-month, 3-month, and 6-month time periods for epilepsy, and (b) differentiating successful treatment and tumor recurrence at 24- hour, 2-month, 4-month, 6-month, 9-month, and 11-month post-LITT for GBM studies. Fig. 9(a) illustrates that T1w MRI and T2-GRE were most discriminative in distinguishing patients with successful treatment and tumor recurrence across all time-points, as compared to T2w MRI, and FLAIR. Similarly, Fig. 9(b) suggest that ADC was identified as being most reflective of early treatment changes (up to 3-months), while T1w was found to be more reflective of early delayed treatment changes (1-month, 3-months) compared to the other protocols under evaluation.

Fig. 9(a) illustrates the contributions of each of the protocols, based on the voting scheme, described in the Methods Section for the epilepsy cohort. The weights of the individual MRI markers were normalized between 0 and 1 over the two studies for different time points. The top 25 profiles with highest mean intensity differences for 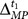 and lowest mean intensity differences for 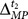, 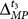, 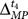 were identified (based on the trends observed in Fig. 5(j)), and the protocols that most commonly occurred over the top 25 weight profiles were identified as candidate MR markers.

Fig. 9(a) illustrates that, ADC was identified as being most reflective of early treatment changes (up to 3-months), while T1w was found to be more reflective of early delayed treatment changes (1-month, 3-months) compared to the other protocols under evaluation. T2 and FLAIR appear to be more discriminative in identifying late treatment changes (around 6-months post-LITT) as compared to ADC, GRE, and FLAIR, while GRE is only nominally reflective of treatment changes at any follow-up time-point post-LITT.

Fig. 9(b) illustrates the contributions of each of the protocol in distinguishing successful treatment and tumor recurrence based on the trends identified in Fig. 8. For each time-point, every MRI protocol was differentially weighted based on their ability to distinguish the two classes (successful treatment and tumor recurrence). The trends in Fig. 8 observed over 6 GBM studies suggest that T1w MRI and T2-GRE were most discriminative in distinguishing patients with tumor recurrence and successful treatment across all time-points, as compared to T2w MRI, and FLAIR. T2w MRI was more discriminative of the two classes after 16-weeks post-treatment. FLAIR, compared to T1w, T2w, and GRE performed poorly in distinguishing the two classes across all time-points.

**Figure 10:**
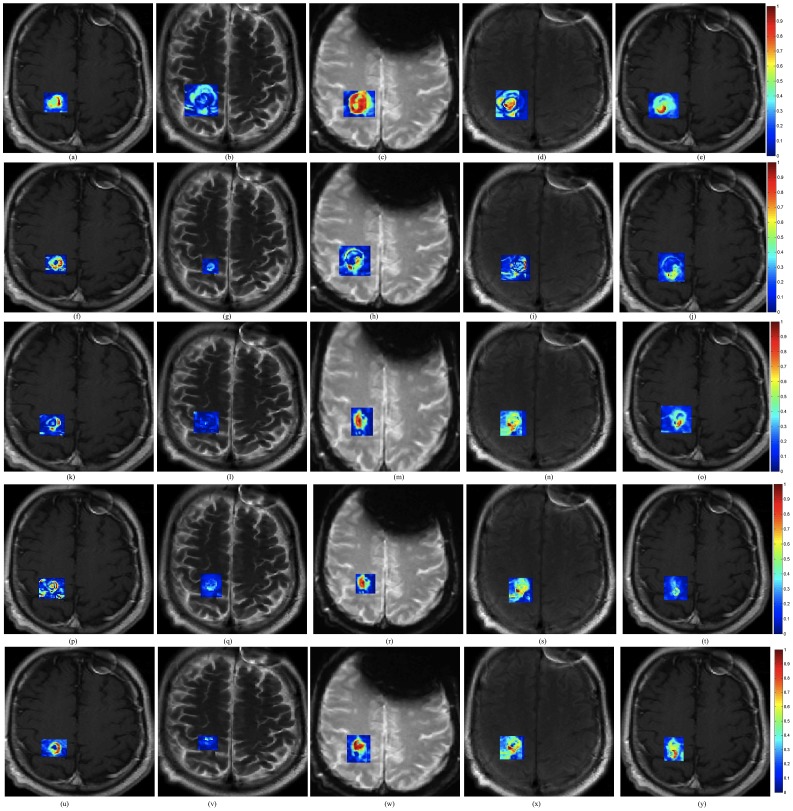
(a)–(e) Difference maps between 24-hours post-LITT and pre-LITT (baseline) for T1w (a), T2w (b), FLAIR (c), and GRE (d). Figs. 10(f)–(i) show difference maps for 2-month post-LITT with respect to baseline, (k)–(n) show difference maps for 7-month post-LITT, (p)–(s) show difference maps for 9-month post-LITT and (u)–(x) show difference maps for 11-month post-LITT with respect to baseline for T1w, T2w, GRE and FLAIR respectively. Corresponding fused multi-parametric MRI maps obtained by weighted combination of individual MRI protocols are shown in Figs. 10(j), (o), (t), (y), corresponding to differences at 24-hour, 2-months, 7-months, 9-months, and 11-months with respect to baseline scan respectively.

Fig. 10 shows corresponding difference maps 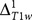 (a), 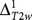 (b), 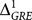 (d) and 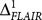 (e) for each of the different MRI protocols obtained for a GBM study. Similar difference maps for each of the protocols for image intensity differences at 2-month with respect to baseline, at 7-months with respect to baseline, at 9-months with respect to baseline and at 11-months with respect to baseline are shown in Figs. 10(f)–(i), (k)–(n), (p)–(s), and (u)–(x) respectively. Figs. 10(j), (o), (t), and (y) show the corresponding fused MP-MRI difference maps obtained as a weighted combination of imaging markers as illustrated in Fig. 9(b). Corresponding temporal MP-MRI profiles for successful (shown in green) and unsuccessful treatment (shown in red) obtained by the optimal weights is shown in Fig. 8(f). Note how different MRI protocols change over time post-LITT and contribute to creating a fused MP-MRI profile that is more discriminative of successful treatment and tumor recurrence for GBM studies, as compared to individual MRI protocols (Fig. 8(a), (b), and (c)). Fig. 8(e) shows a trend of volumetric changes over time (obtained on T1w MRI). Please note the similarity in trends obtained via volumetric changes (state-of-the-art) with that obtained from plotting changes on per-pixel basis on multi-parametric MRI profile.

## Discussion

LITT holds tremendous potential as a minimally invasive treatment modality for clinical applications such as epilepsy [3], [16] and glioblastoma multiforme [1], [2]. A significant advantage enjoyed by LITT is that since it is based on thermal destruction of the target and not constrained by a maximum dose limit, it may be used opportunistically multiple times post-treatment if required [5]. Although highly promising, there is relatively little information regarding the specific *in vivo* imaging characteristics of LITT-induced changes for neurological disorders. In this work, we developed a quantitative framework to identify MRI markers that evaluated early and delayed changes in MRI markers due to LITT.

Only few studies so far have investigated the efficacy of LITT in the context of GBM and epilepsy. Schulze et al. [6] characterized the underlying laser-tissue interactions and resulting histological changes as central coagulation necrosis and peripheral edema as early changes, and subsequent resorptive changes, the formation of a rim of granulation tissue as delayed-changes, post-LITT for GBM studies. Schwabe et al. [7] similarly attempted to characterize treatment related changes in T1 and phase maps for monitoring effects of LITT for GBM. Similarly, Curry et al. [4] in a recent study on a small cohort of 5 epilepsy patients investigated the effect of LITT by monitoring volumetric changes in MRI. Another recent study by Carpentier et al. [32] evaluated four GBM patients that had recurrent tumors after surgical resection. The study reported that LITT can be efficient as a salvage therapy to improve patient survival by a few weeks. The recent results of first in-human Phase I clinical trial [33] using LITT for recurrent GBM demonstrated the utility of delivering controlled laser ablation for improving patient survival in GBM patients. The closest work to this study was recently published by Danish et al. [3], where the trends in volumetric changes on contrast-enhanced T1w MRI over time post-LITT recurrent metastatic tumors patients were studied. However, to our knowledge, none of these studies have investigated per-voxel quantitative changes in multi-parametric MRI markers over time to study early effects of LITT on the tumor lesion, as well as identify MRI markers that are more sensitive to capturing LITT-related effects. The temporal analysis of changes in MR markers can further allow for a more systematic and complementary analysis of patient's response to LITT by evaluating localized per-voxel changes as against measuring a global volume parameter.

The quantitative framework presented in this work was employed to interrogate the following effects. (1) Evaluating temporal profiles of changes in MRI markers over-time, (2) identifying which post-LITT MRI marker changes most dramatically over time and is hence more sensitive to capturing treatment changes over time, and (3) identifying when LITT induced changes have subsided allowing for assessment of treatment efficacy. In the first objective, changes in MRI markers over time were monitored and compared from patients with no signs of recurrence to patients with disease recurrence. Schwabe et al. [7] have demonstrated that significant changes in MR markers subside over time for patients with successful treatment as against patients with tumor recurrence where the changes either remain prominent or resurface over time. Based on the findings of Schwabe et al. [7] we assumed that the trends of changes in MR imaging markers across successful LITT treatment and tumor recurrence may be significantly distinct. Additionally, Schulze et al. and Schwabe et al. have demonstrated the presence of a rim effect as a benign effect of LITT treatment for GBM due to deposition of necrotic tissue around the ablation zone. Our findings are aligned with these findings as the halo effect was found to be accentuated in the difference heat maps Figs. 6 (f), (g), (h), and (i) as compared to the original images (Figs. 6(a), (b), (c), (d), and (e)). A similar Halo effect was also observed in heat maps obtained on the epilepsy cohort (Fig. 5(f), (g), (h) and (i)). Similarly, based on clinical findings in Curry et al. [4], the exaggerated changes in MP-MRI markers for epilepsy studies during the first one month as shown in Fig. 7 may be attributed to edema and swelling caused due to the LITT treatment.

Towards the second objective, candidate MR imaging markers from multi parametric MRI that better captured different treatment related changes post-LITT were identified. Our preliminary analysis on 6 GBM studies suggested that T1w MRI and T2-GRE may be most discriminative in distinguishing patients with successful treatment and tumor recurrence across all time-points, as compared to T2w MRI, and FLAIR. Similarly, our analysis on 4 epilepsy patients suggested that ADC may be more reflective of early treatment changes (up to 3-months), while T1w may be more reflective of early delayed treatment changes (1-month, 3-months) compared to the other protocols under evaluation. A weighted temporal profile was created via a combination of these candidate MRI markers, where weights are computed based on the ability of MR markers to distinguish successful LITT treatment and tumor recurrence. Most of the existing strategies on evaluating treatment changes post-treatment have so far only explored changes in individual MR imaging markers. For e.g. in a recent study involving head and neck cancers, King et al. [17] evaluated changes in T2-w MR intensities to study early post-chemo-RT (CRT) assessment of the primary tumor. The study demonstrated the efficacy of T2-w MRI in accurately localizing early CRT changes within 8-weeks post-CRT. Chan et al. [34] studied morphological characteristics of late radiation induced changes on T1w spin echo, T2-w spin-echo, GRE, FLAIR and T1w post-contrast sequences demonstrating that the late effects of radiation are more varied on different MRI protocols than have been reported in the literature. Khayal et al. [35] investigated changes in diffusion parameters at pre-, mid-, and post-RT for post-surgical GBM patients to identify imaging markers that correspond to long-term patient survival.

The third and final objective of this work was to identify when LITT induced changes subside for assessment of treatment efficacy. This analysis could provide clinicians with clinical insights to more carefully monitor changes in imaging markers at the optimal time point at which early treatment changes subside and changes due to tumor recurrence may become discernible. In a related study involving prostate cancer, Foltz et al. [36] evaluated short- term treatment changes by evaluating changes in values of ADC and T2 relaxation every 2 weeks with respect to baseline-MRI, over the course of 8 weeks. The significant findings of the work by Foltz et al. included, (a) identifying MRI marker changes that were found to be correlated to specific treatment changes, and (b) identifying an optimal time-point since initiation of treatment, when treatment failed (known as biochemical failure. Similarly, a recent study by Danish et al. [3] on evaluating trends of volumetric changes in recurrent metastatic tumors post-LITT reported about intracranial lesions be made at least 24-hours after treatment rather than immediately after LITT. The study also demonstrated the increase in volume at 24-hours, which is in alignment with the findings reported in this work (Figs. 5, 6 and 7).

To summarize, our preliminary analysis on four epilepsy patient studies suggested that (a) LITT related changes appeared to dissipate in a period of 4 weeks following LITT, and (b) ADC may better capture early treatment changes (up to 3-months), while T1w may better capture early delayed treatment changes (1-month, 3-months), compared to the other MRI protocols under evaluation. These findings although limited to four studies appear to provide some initial insights on the ability of the different MR protocols in evaluating treatment changes at different time-points. Similarly, our preliminary results on a cohort of six GBM studies evaluated in this work suggested that, (a) trends of changes in MRI markers may serve as potential surrogate markers to distinguish patients with continued control from the ones with disease recurrence, and (b) T1w MRI and T2-GRE may be better markers for early disease recurrence, as compared to T2w MRI and FLAIR.

We do acknowledge a few limitations of the current work. Only a small number of GBM patients were analyzed. These patients were all at different time points in their disease, and many known variables important for tumor control are not accounted for in this study. Three of the cases presented for treatment very late in their disease process, making any concrete conclusions about the correlation of changes with treatment response difficult. There may be an inherent selection bias in this small cohort, as the patients who survive their primary and secondary treatments and remain in a capacity to undergo this treatment, have already proven to be long survivors after diagnosis. The issue of the “crucial time point” when treatment related changes subside will need more in depth analysis with a larger group of patients, and a greater understanding of the already well-established prognostic indicators such as age, extent of resection, and MGMT methylation status. Since multiple MRIs at the same time on the same patient were not available, our framework could not be evaluated against a control population set. Additionally, per-voxel changes evaluated in this work were not correlated with extent of thermal dose, tumor morphology, shape, volume changes, and probe placement, which will a part of future work.

In the absence of sufficient patient data, the temporal profiles developed in this work could not be validated within a prognostic classifier, and hence its efficacy in capturing patient's response to treatment remains to be verified in a future study. However, it is important to remember that the goal of this work was primarily to develop a quantitative framework to evaluate voxel-by-voxel changes in MR markers over time for identification of early treatment changes post-LITT.
